# The Importance of Thrombin in Cerebral Injury and Disease

**DOI:** 10.3390/ijms17010084

**Published:** 2016-01-11

**Authors:** Harald Krenzlin, Viola Lorenz, Sven Danckwardt, Oliver Kempski, Beat Alessandri

**Affiliations:** 1 Institute for Neurosurgical Pathophysiology, University Medicine Mainz, Langenbeckstr. 1, Mainz 55101, Germany; viola.lorenz@childrens.harvard.edu (V.L.); oliver.kempski@unimedizin-mainz.de (O.K.); Beat.Alessandri@unimedizin-mainz.de (B.A.); 2 Clinic for Neurosurgery, HELIOS Dr. Horst Schmidt Clinic (HSK) Wiesbaden, Ludwig-Erhard-Strasse 100, Wiesbaden 65197, Germany; 3 Division of Newborn Medicine, Boston Children’s Hospital, 300 Longwood Avenue, Boston, MA 02115, USA; 4 German Centre for Cardiovascular Research (DZHK), University Medicine Mainz, Langenbeckstr. 1, Mainz 55101, Germany; Sven.Danckwardt@unimedizin-mainz.de; 5 Institute for Clinical Chemistry and Laboratory Medicine University, Medical Center Mainz, Langenbeckstr. 1, Mainz 55101, Germany; 6 GCenter for Thrombosis and Hemostasis (CTH), University Medical Center Mainz, Langenbeckstr. 1, Mainz 55101, Germany

**Keywords:** thrombin, prothrombin, cerebral thrombin system, Parkinson’s disease, Alzheimer’s disease, multiple sclerosis, stroke, intracerebral haemorrhage

## Abstract

There is increasing evidence that prothrombin and its active derivative thrombin are expressed locally in the central nervous system. So far, little is known about the physiological and pathophysiological functions exerted by thrombin in the human brain. Extra-hepatic prothrombin expression has been identified in neuronal cells and astrocytes via mRNA measurement. The actual amount of brain derived prothrombin is expected to be 1% or less compared to that in the liver. The role in brain injury depends upon its concentration, as higher amounts cause neuroinflammation and apoptosis, while lower concentrations might even be cytoprotective. Its involvement in numerous diseases like Alzheimer’s, multiple sclerosis, cerebral ischemia and haemorrhage is becoming increasingly clear. This review focuses on elucidation of the cerebral thrombin expression, local generation and its role in injury and disease of the central nervous system.

## 1. Introduction

Thrombin is a trypsin-like allosteric serine protease active within the coagulation cascade. It belongs to the chymotrypsin family and is generated by proteolytic cleavage of its inactive precursor prothrombin by the prothrombinase complex. This activation complex consists of activated factor V (FVa), FX (Stuart-Prower factor), Ca^2+^ and phospohlipids [1,2]. Thrombin mediates the conversion of fibrinogen to fibrin, the main constituent of a blood clot. Furthermore, thrombin activates the blood coagulation factors V, VIII, XI, XIII and the anticoagulant protein C [3].

Beside the central role in the coagulation cascade, the generation of thrombin leads to receptor mediated inflammatory responses, cell proliferation/modulation, cell protection and apoptosis [4,5]. The irreversible inactivation of thrombin *in vivo* is mainly mediated by the serpin (serine protease inhibitor) antithrombin (AT). Other thrombin inhibitors, including hirudin and heparin, bind to either of two substrate binding sites (exosites) [6]. Thrombin consists of a light A-chain and a heavy B-chain with two extended exosites for increased substrate affinity. Exosite I recognises fibrinogen, fibrin, FVa, thrombomodulin and hirudin, while exosite II is responsible for heparin, platelet integrin membrane receptors such as glycoprotein Ibα (GPIbα) and glycosaminoglycan binding [7,8]. Thus, thrombin acts as a polyfunctional signalling molecule binding to several substrates with a broad structural diversity [9].

The vast majority of prothrombin is produced in the liver and released into the plasma. It circulates within the bloodstream until it is converted into mature thrombin in the the coagulation cascade [10]. Thrombin is a large, spherical molecule, with a major groove around its equatorial axis, that is unable to pass the blood-brain barrier (BBB) [9]. In cases of a BBB breakdown, e.g., during head trauma, severe epilepsy, inflammation and other pathologic conditions, thrombin enters the brain and reaches high concentrations [11]. Yet, thrombin has been demonstrated within the central nervous system (CNS) in rat and human specimens under physiological conditions [12]. Additionally, main thrombin regulatory factors such as FX, protease nexin-1 (PN-1), AT III and thrombin-activated receptors have been identified in these specimens, indicating a potential role of thrombin in the CNS [13].

Prothrombin has been found on neuronal cells and astrocytes via mRNA measurement in rat and human nervous system tissue, but the actual amount of brain derived prothrombin is expected to be 1% or less compared to hepatic concentrations [14]. Prothrombin has been found in the olfactory bulb, cortex, colliculus superior and inferior, corpus striatum, thalamus and hippocampus in rat brain [14]. Almost all regions express prothrombin transcripts except for white matter areas [14]. The physiological importance of this brain-derived generation is mostly unknown. It has been found that the expression of prothrombin mRNA follows a developmental pattern with a strong increase post partum in the rat brain [15]. In later stages, during adolescence, neuronal cells express the prothrombinase complex. At this time point, FX immune-reactivity was found in microglia and brainstem neurons [16,17].

Besides the involvement of thrombin in developmental processes, there has been evidence of various other functions within the CNS, exerted in a dosage dependent manner [18,19]. At low concentrations thrombin causes neuron and astrocyte modification, induces glial cell proliferation and exerts a neuroprotective influence [18]. At high concentrations, a neurotoxic effect with disruption of the BBB, oedema and inflammation, has been reported [20]. Thrombin inhibitors such as PN-1, AT III, a1-antitrypsin, a2-macroglobulin, C1-inhibitor and thrombomodulin are also locally expressed in the brain [12,21,22,23,24,25]. In order to control undesired thrombin effects in case of a BBB breakdown, PN-1 is predominantly expressed around the intraparenchymal blood vessels [26].

This local expression of prothrombin activators and inhibitors in the CNS (in the presence of an intact BBB) suggests that the neuronal prothrombin, expressed in neurons and glia cells, might be the primary source of the brain-derived thrombin and might reflect its yet not fully understood physiological significance [12,23,27]. This reviews aim is to outline the available literature and to shed light onto possible functions in health and disease.

## 2. Thrombin Signalling in Health and Disease

The serine protease thrombin exerts its physiological function through soluble target proteins and G-protein-coupled receptors. These protease-activated receptors (PARs) belong to a family of seven transmembrane domain receptors, activated through a di-phasic cleavage process of the extracellular N-terminus [28,29]. The PAR receptor family consists of 4 members so far. PAR-1, PAR-3 and PAR-4 are assumed to interact with thrombin, while PAR-2 is activated by other serine proteases such as trypsin and mast cell tryptase [30,31,32,33]. Depending on each cell type, PAR-1 is coupled to heterotrimeric G proteins including pertussis toxin (PTX)-sensitive G proteins (Gi/Go) and/or PTX-insensitive G proteins (Gq) [18,34]. Intracellular signal transduction via activation of phospholipase C (PLC), generation of inositol trisphosphate and diacylglycerol leads to consecutive Ca^2+^ release and protein kinase C (PKC) activation. Further activation mechanisms via non-receptor tyrosine kinases like Src and focal adhesion kinase, rat sarcoma protein (Ras) and Ras-related protein Rho, activation of phosphatidylinositol 3-kinase (PI 3-kinase) and mitogen- activated protein kinase (MAPK) have been reported [35]. While PARs are well characterized within the blood vascular system, their importance in the CNS is not completely understood. PARs are expressed throughout the brain in neurons, microglia, astro- and oligodendrocytes [36,37].

PAR-1 and prothrombin mRNA expression are co-localized and follow similar developmental patterns in rat brain [38]. PAR-1 mRNA first occurs in the late embryonic and early postnatal nervous system. It becomes more abundantly expressed in the adult brain [13]. The intriguing pattern of prothrombin mRNA and PAR-1 expression throughout the pre- and postnatal period suggests a role for thrombin in neuronal development [15]. PAR-1 is abundant in the pyramidal cell layers of the hippocampus, while strong PAR-3 and PAR-4 expression is observed in all cortical layers and the thalamus in rat brains [36]. Multiple receptor subtypes of the PAR family coexist in neuronal cells. In rat neurons and astrocytes all 4 PAR receptors are present [34,39]. The simultaneous expression of prothrombin, FX, PAR-1 and PN-1 in intact nervous tissue suggests a PAR mediated thrombin effect under physiological conditions. However, the thrombin effect is more dominant when blood-derived factors are supplemented, e.g., under conditions when the BBB is disrupted [40].

Increasing evidence has surfaced that thrombin, mainly via PAR-1 activation, influences neurons and glia cells. Although thrombin uses PAR-1, PAR-3 and PAR-4 for signal transduction, it appears that modification and proliferation are predominantly mediated via PAR-1 activation. Thrombin changes the cell morphology of astrocytes, fetal neurons and neuroblastoma cells, where it causes neurite retraction and reverse stellation [41,42,43]. At times, thrombin induced shape changes involve the activation of Rho GTPases, which link plasma membrane receptors to the organization of the actin cytoskeleton [43,44]. Furthermore, thrombin is considered a strong mitogenic agent, exerting its function on astrocytes and microglia through PAR-1 activated tyrosine kinase activity [34,45,46,47]. Cellular proliferation and differentiation signals are regulated through MAPKs such as extracellular signal-regulated protein kinase 1 (ERK1, p44 MAPK) and ERK2 (p42 MAPK) [48]. Further pathways involve the PI 3-kinase pathway and the PLC/Ca^2+^/PKC pathway, however these signalling cascades appear to be cell type specific [18]. Cytoskeletal rearrangements are necessary for growth cone guidance, cell migration and neuronal plasticity. Beyond solely morphological changes, thrombin mediates nerve growth factor and endothelin-1 synthesis, which causes astrocyte proliferation [42,43,44,49,50].

In contrast to neuronal plasticity and regeneration, the occurrence of astrogliosis, in response to a proliferation stimulus, is common in both head trauma and neurodegenerative disease [51]. The activation of PARs is also known to potentiate *N*-methyl-d-aspartate (NMDA) receptor responses, such as glutamate-mediated cell death or provoke post traumatic seizures [52,53]. In exerting a direct influence on astrocyte shape and function, thrombin might contribute to maintaining the BBB integrity and proper brain function.

A cytokine-induced nitric oxide (NO) release and the increased expression of inducible nitric oxide synthase (iNOS) in the presence of thrombin, indicates a possible involvement of a PAR activated pathway in brain inflammation [54,55]. This assumption is further strengthened by the finding that PARs are expressed in microglial cells activated in the wake of brain injury and stimulates their proliferation [56]. Microglia constitute the innate immune system of the central nervous system and represent cellular mediators of neuroinflammatory processes. As thrombin is capable of activating glia cells, including astrocytes and microglia, *in vitro* and *in vivo*, it has long been considered a proinflammatory agent and is associated with glia scar formation [34,39,46,57]. In microglia p38 MAPK and c-Jun N-terminal kinase (JNK) are activated by thrombin. Microglial activation involves PAR-1, PAR-3 and PAR-4 [46,57]. Thrombin facilitates the release of cyclooxygenase 2 (COX-2), iNOS, tumor necrosis factor-α (TNF-α), interleukin-1α/β (IL-1α/β), IL-6, IL-12, and boosts CD40 expression, all potent pro-inflammatory factors [46]. It has been shown that COX-2 and iNOS expression by microglial cells causes cell death of dopaminergic neurons in the substantia nigra of rat brains. Cell death is averted by iNOS, COX-2 (inhibitor DuP-697) or MAPKs inhibition [58,59]. Astrocytes, likewise activated by thrombin, are known to act as immune effector cells alongside the microglia through release of further proinflammatory mediators such as arachidonic acid, the chemokine growth-regulated oncogene/cytokine-induced neutrophil chemoattractant-1 (GRO/CINC-1), IL-8 and NO [60,61]. Additionally, IL-6, IL-1α/β and TNF-α mRNA expression, exaggerating the proinflammatory environment has been reported in murine glioma cells [55]. Conversely, these cytokines might be capable of a prothrombin activation themselves [62].

In the light of these findings an ever-increasing role of thrombin in health and disease in our brain becomes evident. In neurodegenerative diseases such as Alzheimer’s disease (AD) thrombin has been found in the neurofibrillary tangles (NFTs) and senile plaques of affected patients [63]. Microglia activation is not solely mediated via PAR and is not triggered by PAR specific agonists [64]. Furthermore, elevated thrombin levels of the CNS have not only been linked to neurodegenerative diseases, but also to multiple sclerosis (MS), human immunodeficiency virus (HIV)-related dementia (HIVD) and Parkinson’s disease (PD). In addition to its impact on many neuroinflammatory and neurodegenerative diseases, experiments using organotypic slice cultures demonstrate that thrombin exerts influence on oedema formation and the amount of secondary damage after cerebral ischemia and haemorrhage. This influence is exerted in a dosage dependent manner [65]. Paradoxically, endogenous neuroprotective mechanisms can be triggered by intracerebral infusion of a low dose of thrombin (thrombin preconditioning (TPC)), which fails to produce marked brain oedema by itself, leading to tolerance to cerebral ischemia or haemorrhage [65,66]. Thus, the modulation of thrombin activity has become of increasing interest in the quest for new therapeutic targets. The following chapters seek to elucidate the contemporary knowledge of these links between thrombin and ischemic, neuroinflammatory and neurodegenerative diseases that affect our central nervous system.

## 3. Thrombin in Neurodegenerative Disease

**Figure 1:**
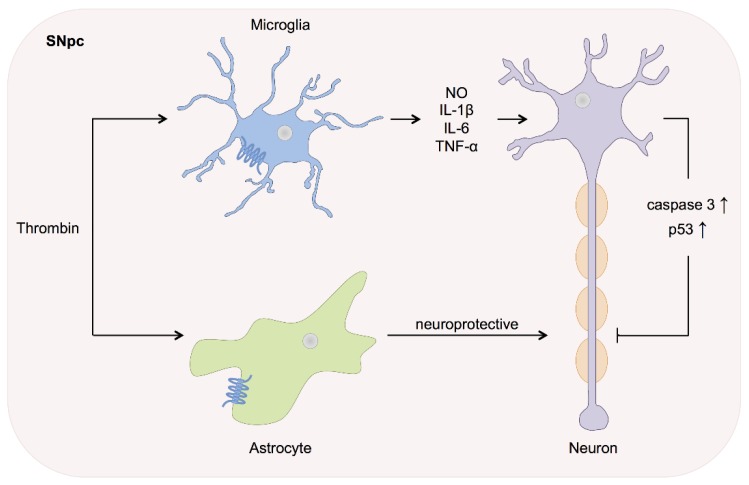
Parkinson’s disease. In PD a progressive loss of dopaminergic neurons and microglia activation in the substantia nigra pars compact (SNpc), is characteristic. Microglia activation by thrombin leads to the expression of NO, IL-1β, IL-6 and TNF-α, which causes a release of caspase-3 and p53 from the dopaminergic neurons and results in neuronal death. On the other hand, PAR-1 activation in astrocytes seems to exert a more neuroprotective influence.

Parkinson’s disease*:* PD is characterized by a progressive loss of dopaminergic neurons within the substantia nigra pars compacta (SNpc) as well as a microglia activation [67]. Increasingly, thrombin derived from blood or brain prothrombin has been associated with injury modulation and progression in PD. This influence seems likewise dose dependent, as described in other pathological conditions and increases with higher concentrations (~20U) [68]. In brain slices of diseased patients, prothrombin and PAR-1 protein are up-regulated in glial fibrillary acidic protein (GFAP) positive astrocytes. Also, thrombin is increased in the vessel wall within the SNpc [69]. Studies demonstrated thrombin induced dopaminergic cell loss *in vitro* and a decrease of dopaminergic neurons after thrombin injection with the substantia nigra *in vitro* [58,68,70,71]. The key histo-pathological characteristics of PD, neuroinflammation and oxidative stress, were observed after thrombin injection into the substantia nigra [72]. Microglia activation by thrombin led to expression of proinflammatory factors such as NO, IL-1β, IL-6 and TNF-α, resembling the pathophysiology of PD (Figure 1). The pro-apoptotic proteins caspase-3 and p53 are similarly up-regulated in nigral dopaminergic neurons [58,71,72,73]. Thus, microglia activation by thrombin lead to neuronal death *in vivo* and *in vitro*, mimicking pathophysiological changes likewise observed in PD [74]. Intriguingly, despite the pro-inflammatory capacity of the PAR-1 pathway, thrombin appears to exert its neurotoxic effects on dopaminergic neurons in a PAR-1 independent manner [73]. Furthermore, PAR-1 activation seems to possess neuroprotective effects in rat models. PAR-1 agonists mimic these effects, while PAR-1 antagonists increase neurological deficits secondary to 6-hydroxydopamine (6-OHDA) administration [75,76]. As PAR-1 is indeed up-regulated on astrocytes within the SNpc in PD patients, but seemingly absent from microglia, a neuroprotective influence appears more likely and might outweigh a possible neurotoxic capacity.

In contrast, other studies demonstrate the constitutive expression of PAR-1 on murine microglia [46]. The expression of PAR-1 is discussed as a restorative measurement taken by the brain to provide neuroprotection against neuronal degeneration and cell death of dopaminergic neurons, caused by noxious insults during the progression of PD pathology [69]. At the same time, distant thrombin effects might be held accountable for other detrimental processes, as it has been found in rat brain [77]. Similar to the findings observed in ischemic stroke, mild TPC and, much more surprising, delayed thrombin infusion, protects against 6-OHDA induced cell damage in PD models [78]. The preconditioning effects were abolished in animals previously treated with PAR-1 antagonists. In contrast to these findings, neuronal damage and resulting behavioural deficits were much more pronounced, if thrombin or PAR-1 agonists were simultaneously applied with 6-OHDA [78]. These findings stress the importance of time and concentration of thrombin occurrence in terms of its pathophysiological implication. Furthermore, it is noteworthy, that Parkinson like-effects such as tremors, chorea or asterixis, which are responsive to L-dopamine, have been observed in patients which previously suffered from intracerebral haemorrhage with consecutive thrombin influx at the time of ictus [79]. Thus, haemorrhages near the nigrostriatal tract might represent a potential way in which thrombin exerts influence on PD. Furthermore, the activation of microglia, in a yet not fully understood manner, might lead to Ca^2+^ triggered NO synthesis, contributing to the degeneration of dopaminergic neurons *in vivo* [58,78].

**Figure 2:**
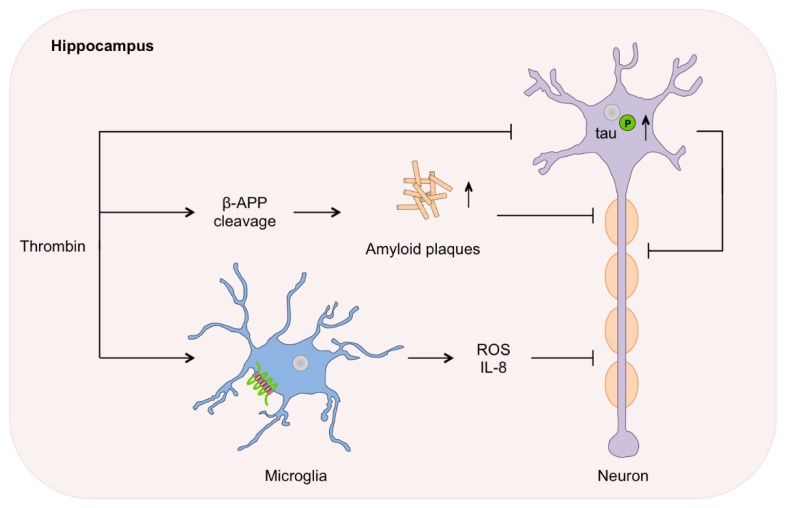
Alzheimer’s disease. In brains of patients with AD, thrombin is present in senile plaques, amyloid deposits, microvessels and neurofibrillary tangles. Thrombin-dependent activation of PAR1/4 results in hyperphosphorylation and aggregation of tau protein leading to apoptosis of predominantly hippocampal neurons. Furthermore, thrombin induces the cleavage of amyloid-β precursor protein (β-APP) leading to the amyloid β (Aβ) accumulation (amyloid plaques), which have neurotoxic capacity. Additionally, microglia activation by thrombin leads to the generation of reactive oxygen species (ROS) and proinflammatory proteins e.g., IL-8.

Alzheimer’s disease: Thrombin was first associated with the pathophysiology of AD in 1992 [63]. The hallmarks of AD comprise the accumulation of intracellular tau protein aggregates and the occurrence of neurotoxic amyloid beta (Aβ). Thrombin is present in senile plaques, amyloid deposits, microvessels and NFTs in brains of patients with AD. Prothrombin mRNA is expressed in neuronal cells [27,80]. In a rat model for AD, PAR-1, and to a lesser extent PAR-3 and PAR-4, are up-regulated in the hippocampus [81]. The finding, that the activity of the thrombin inhibitor PN-1 is reduced due to thrombin-PN-1 complex formation, falls in line with the previously mentioned results [82]. Thrombin is known to proteolyze the microtubule-associated tau protein *in vitro*, but fails to process phosphorylated tau protein, causing intracellular aggregates in hippocampal neurons [83]. The activation of PAR-1/4 and the ERK1/2 pathway is involved in thrombin induced hyperphosphorylation and tau aggregation [57,83]. These tau deposits have been shown to be neurotoxic leading to apoptosis of predominantly hippocampal neurons (Figure 2) [83]. Thrombin further leads to Aβ accumulation (amyloid plaques) by cleavage of amyloid-β precursor protein (β-APP) *in vitro* [54,84]. The neurotoxic capacity of Aβ is further increased by thrombin via intracellular Ca^2+^ influx and increase of oxidative stress, while other reports suggest that thrombin might alleviate the neurotoxic Aβ effect [85,86]. It is worth to mention that PN-1 seems to protect neurons against Aβ induced neurotoxicity [86].

Further effects of thrombin lead to generation of reactive oxygen species (ROS) and the expression of proinflammatory proteins such as IL-8 and integrins. The production of ROS is triggered via activation of microglial NADPH oxidase, while the expression of proinflammatory proteins is a further source of oxidative stress and cell death *in vitro* [80,87]. Both, oxidative stress and inflammation, are strongly associated with the pathomechanisms in AD and therefore, further strengthen the connection between the pro-neuroinflammatory agent thrombin and this disease. Intriguingly, thrombin itself is released from brain endothelia cells under pathological conditions [80]. The transcription factor hypoxia inducing factor 1α (HIF-1α) is constitutionally up-regulated in brains with AD and decreases after direct thrombin inhibition (e.g., dabigatran) [80,88,89]. Nevertheless, all these interrelations of thrombin, PAR and the accumulation of tau, were so far only shown *in vitro.* In rats, intracerebral administration of thrombin increased the levels of apolipoprotein E that facilitates β-amyloid deposition and cognitive deficits [90]. The involvement of thrombin in the formation of amyloid plagues, NFTs and neuronal loss remains to be proven in patients with Alzheimer’s disease.

**Figure 3:**
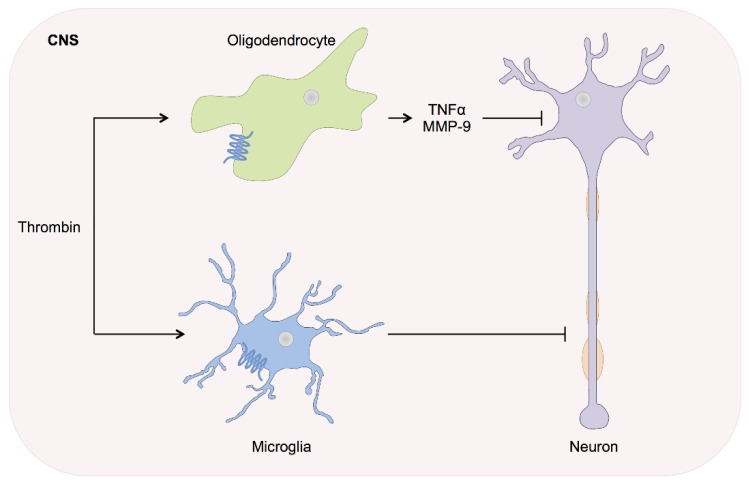
Multiple sclerosis. In MS fibrin deposits are related to axonal damage and demyelination. Thrombin-dependent PAR-1 activation on oligodendrocytes mediates the release of proinflammatory factors such as TNF-α or matrix metalloproteinase-9 (MMP-9). Furthermore, increased thrombin activity in the spinal cord was associated with increased microglia activation and neuronal demyelination, which correlate with a disease progression and neurological deficits.

Multiple sclerosis: MS is a neurological disease in which inflammation and demyelination lead to progressive disability. The consecutive axonal loss is associated with the activation of the coagulation cascade and accumulation of fibrin in the cerebral vasculature in animal models [91]. Experimental autoimmune encephalitis (EAE) models in mice demonstrated that fibrin depositions correlate with axonal damage and demyelination [92,93]. Although thrombin inhibitors and thrombin-AT III complexes are elevated in the EAE, no elevation of thrombin has been found in the CNS of MS patients [94,95]. At the peak of EAE, thrombin activity is dramatically increased in the spinal cord, before any motor impairment was apparent. In these lesions, a strong correlation between thrombin activity and fibrin, increased microglial activation and the extent of demyelination has been described (Figure 3) [96]. Thrombin activation begins early at disease onset, prior to demyelination, and correlates with disease progression. This strong association between thrombin activity and BBB disruption, microglial activation, inflammatory demyelination, and axonal damage predestines thrombin as a putative disease marker in neuroinflammation and MS [96].

Oligodendrocytes express PAR-1 receptors, which have been previously demonstrated to mediate proinflammatory factor expression after activation by thrombin [97]. Thus, they might play an important role in MS pathophysiology. Macrophage associated PAR-2 activation leads to oligodendrocyte death in the EAE animal model [98]. Furthermore, a BBB breakdown has been found in EAE animals in early clinical stages [99]. A recent study confirmed the potential of PAR-1 inhibitors in EAE mice by stabilizing the BBB and by suppressing demyelination and infiltration of inflammatory cells in the spinal cord and brain [100]. These findings were accompanied by a significant reduction of thrombin, TNF-α, as well as a down-regulation of matrix metalloproteinase-9 (MMP-9) expression and preserved expression of occluding/zonula occludens (ZO)-1 in the brain. Analogous results were obtained in astrocyte cultures *in vitro* further strengthening the interrelation of MS and thrombin as well as placing PAR inhibitors in the limelight of pharmacological research [100]. Nevertheless, it remains uncertain if thrombin influx is mediated solely via BBB breakdown or if local expression of prothrombin and consecutive thrombin generation contributes to the pathophysiology of neuroinflammation.

HIV-induced dementia and HIV-associated ischemia: HIV-associated encephalitis and dementia are characterized by neuroinflammation and consecutive neuronal damage. The level of prothrombin protein and mRNA is increased in brains of patients with HIV-induced encephalitis. Likewise, the expression of PAR-1 is up-regulated on astrocytes [94]. PAR-2 is up-regulated on neurons of HIV patients indicating a possible involvement in HIVD [101]. HIVD has been associated with neurite retraction including dendritic simplification and reduced synaptic density, potentially reflecting PAR-induced cell shape change, neurite retraction and/or neurotoxicity. Recent evidence suggests that neuroinflammation in HIV patients might be triggered in a PAR-1-dependent manner and might even influence the viral infectiosity [102]. Peripheral blood effector memory CD4(+) and CD8(+) T-lymphocytes were found to express PAR-1 and this expression is increased in CD8(+) T-cells from HIV-infected patients. Thrombin enhances cytokine secretion in CD8(+) T-cells from both, healthy controls and HIV-infected patients, which ultimately leads to structural changes, including cell polarization, enhanced cytokine gene expression and consecutive release of interferon-γ. Thus, one might speculate, that thrombin mediates the cross-talk between the coagulation system and the adaptive immune system at sites of vascular injury through increased T-cell motility and production of proinflammatory cytokines [103]. To further aggravate the negative thrombin effect in HIV infections, direct regulation of the GP120 epitope and 2F5 antigen on HIV infected cells has been demonstrated [104]. Thus, thrombin might facilitate the HIV-induced cell-fusion rate in a concentration dependent manner. Using computational models, a consistent increase in thrombin and concomitant decrease in key anticoagulants (e.g., lower AT and protein C), during untreated HIV replication, has been demonstrated. In these models, thrombin generation rose by 24% to 48% [105]. Beyond that, HIV infection leads to a breakdown of the BBB which might facilitate the influx of prothrombin/thrombin into the brain and further fostering the above mentioned mechanisms [106]. Despite the increasing data, these findings are still under debate, as some studies claim an unaltered thrombin generation in HIV patients [107]. On a daily clinical level, HIV infection leads to activation of coagulation, with a resulting increased risk for atherosclerosis, venous thromboembolic events and ischemic cerebral infarction [108].

## 4. Thrombin in Stroke and Intracerebral Haemorrhage

Symptoms brought about by a sudden loss of cerebral blood supply, are summarized as stroke. Focal ischemia due to a vascular narrowing or blockage might lead to local hypoperfusion and consecutive stroke symptoms. Possible reasons include vasospasms, thrombosis, and thromboembolic events. Intracerebral haemorrhage (ICH) is a subtype of stroke accounting for approximately 15% of all deaths from stroke [109]. Recent evidence indicates that thrombin plays a modulatory role in traumatic brain injury as well as in ischemic and haemorrhagic stroke.

An increase of thrombin and its precursor prothrombin has been demonstrated in rat brains after focal ischemia [110]. Activated factor X (FXa) leads to consecutive thrombin generation in rat hippocampal slice cultures secondary to oxygen or glucose deprivation [111]. In addition to local thrombin formation, BBB disruption secondary to ischemia and haemorrhage leads to thrombin influx, originating from the bloodstream. In parallel, an up-regulation of PAR-1 and PAR-3 secondary to hypoxia and glucose deprivation (OGD) has been demonstrated in rat hippocampal slices [36]. In contrast, PAR-1 is down-regulated after transient focal ischemia induced through microinjection of the vasoconstrictor endothelin-1. PAR-3 and PAR-4 are regulated inversely in a time dependent manner [112]. Ischemia-dependent PAR-1 and PAR-3 expression were found in microglial cells, while PAR-4 signalling was increased within the penumbra [113]. Despite the regulation of different PAR receptors, the effect of thrombin appears to be largely mediated via PAR-1.

**Figure 4:**
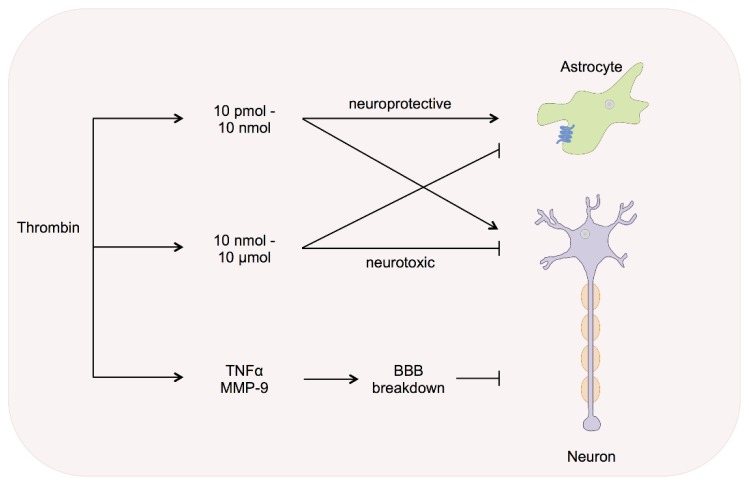
Stroke/intracerebral haemorrhage. Ischemia of brain-tissue leads to an increase in local thrombin and to thrombin influx via BBB breakdown. Thrombin concentrations in pico- to nanomolar range (10 pM–10 nM) are shown to protect hippocampal neurons and astrocytes from a variety of cellular insults. In sharp contrast, for higher concentrations in nano- to micromolar range (10 nM–10 μM) adverse effects, such as increased death of motor neurons and cells of the hippocampus, are documented. Oedema formation after ICH is linked with BBB breakdown and increased thrombin infusion leading to TNF-α and MMP-9 up-regulation. The release of proinflammatory factors contributes to neuroinflammation and neurodegeneration.

The instillation of thrombin concentrations in nanomolar and in micromolar ranges (10 nM–10 μM) induces cell death in hippocampal slice cultures and in motor neurons (Figure 4) [65,114]. In contrast, thrombin protects hippocampal neurons and astrocytes at concentrations within the picomolar to the nanomolar range (10 pM–10 nM) against a variety of cellular insults, such as OGD, hypoglycemia and ROS [51,65]. PN-1 is secreted *in vitro*, but its expression levels remain unchanged under ischemic conditions *in vivo* due to triggered *de novo* synthesis [115]. It is thus hypothesized, that the balance between thrombin and PN-1 might be essential to optimize modulatory and repair processes in stroke [116]. This, together with recent data strongly suggests the influence and importance of thrombin signalling and modulation under ischemic conditions within the CNS.

Increasing data on protective effects of thrombin in pathological conditions such as stroke and haemorrhage becomes available. It was shown that intracerebral thrombin administration (thrombin preconditioning, TPC) leads to oedema reduction in the wake of intracerebral haemorrhage [117]. TPC reduces the infarct volume after cerebral ischemia, whereas Hirudin and PAR-1 antagonists reverse the neuroprotective TPC effect in animal models [118,119,120]. In contrast, other authors describe a neuroprotective effect of the thrombin inhibitor hirudin, with significantly reduced infarct volumes, when instilled after middle cerebral artery occlusion [121]. Extending the morphological phenotype to clinical conditions, TPC enhances the capacity of mice in motor performance tests [122]. It is hypothesized that TPC-induced ceruloplasmin up-regulation might be one possible mechanism in thrombin-induced brain tolerance to cerebral oedema [123]. Another mechanism might involve JNK as their inhibitors reverse the neuroprotective thrombin effects after focal cerebral oedema [122,124]. Adverse effects of thrombin have been documented at 10-fold higher concentrations of about 100 nM. In such high concentrations, thrombin has been shown to increase neuronal death after ischemic conditions or induce cellular damage *de novo* [125]. In slice cultures the administration of thrombin led to delayed neuronal injury [126]. The mechanisms underlying the PAR-1 mediated neurotoxic effects, after cerebral thrombin accumulation, are still unknown.

ICH is associated with severe oedema formation due to BBB breakdown. The formation of brain oedema has been linked to an increase of thrombin during ICH and focal ischemia [19,127,128,129]. Intracerebral infusion of thrombin leads to TNF-α up-regulation and consecutive increase of brain oedema and neurological deficits in mice [129]. Furthermore, thrombin-induced release of pro-inflammatory factors (e.g., TNF-α and NO) contributes to neuroinflammation, neurodegeneration or, to certain degrees, to neuroprotection [46,58,130,131,132]. The neuroprotective effects exerted by thrombin in lower concentrations are compromised by iron-loaded transferrin and lead to larger cerebral oedemas [133]. On the other hand, it has been shown that intracerebral thrombin administration prior to the occurrence of a haemorrhagic lesion, leads to oedema reduction similar to the effects of TPC observed in ischemic stroke [134]. Iron has been established as an independent factor contributing to brain oedema formation after ICH [135]. It has been shown that neurodegeneration and death depends on PAR-1 and MMP-9 activation by thrombin [136]. The latter leads to extracellular matrix degradation and consecutive BBB disruption. MMP-9-deficient mice are less vulnerable to thrombin-induced brain damage [137]. PAR-1-deficient mice are likewise less vulnerable to focal ischemia while PAR-1 activation leads to an increase in infarct volume and neuronal damage [138]. Motor behavioural impairment was less pronounced in PAR-1-deficient mice secondary to focal ischemic events [139]. The activation of PAR-1 leads to amino acid glutamate release from astrocytes and was increased under hypoosmotic *in vitro* conditions [140]. An increase in cerebral glutamate levels after traumatic or ischemic brain injury exerts receptor mediated excitotoxicity and influences synaptic plasticity [141,142]. NMDA receptor increase might contribute to BBB breakdown and is partially reversed by NMDA receptor antagonist MK-801 or the simultaneous presence of thrombin [52,94,143]. Neuronal degeneration under ischemic conditions has been linked to thrombin-induced apoptosis. Cyclin D1 and Cyclin-dependent kinase 4 (CDK 4), two cell cycle proteins, are increased under hypoxic conditions *in vivo* and *in vitro*. CDK 4 leads to the expression of the proapoptotic protein Bim linking cell cycle control to thrombin-induced apoptosis [144,145,146]. As mentioned above, PAR-1 activation leads to astrogliosis via the MAPK pathway and glial scar formation [39,46,61,147]. The occurrence of glial scars poses an obstacle for neuroregeneration and leads to further neuronal loss after ischemia and ICH [148].

It seems that similar receptors and signalling pathways are deployed by thrombin after hypoxia or haemorrhage induced activation leading to both, neuroprotective and neurotoxic effects, in a time and dosage dependent manner.

## 5. Thrombin Inhibitors in Cerebral Injury and Disease

**Figure 5:**
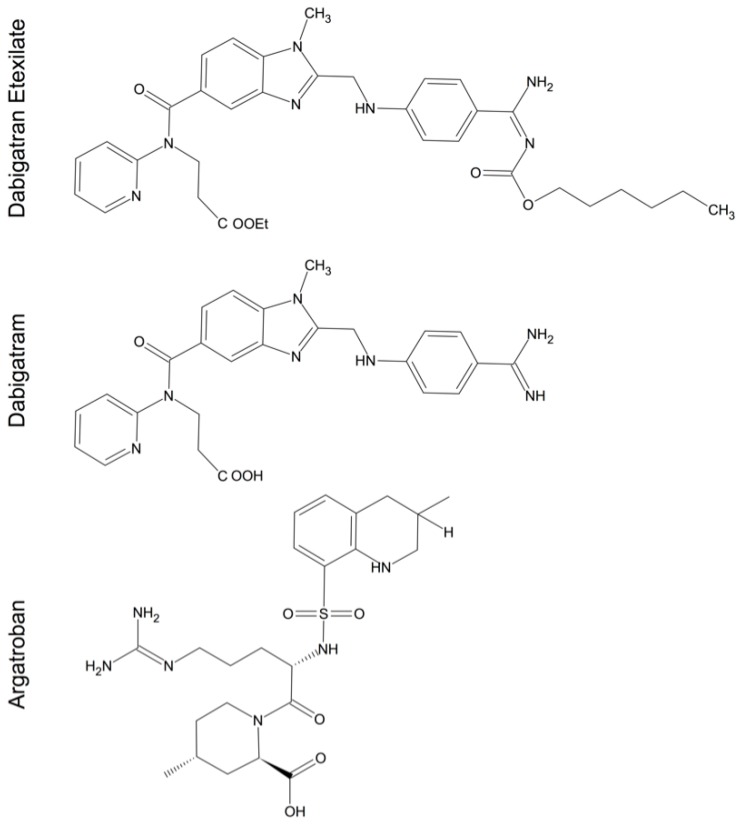
2D chemical structure of common direct acting thrombin inhibitors (DIT). The given structures depict the low molecular weight DTIs: the prodrug dabigatran etexilate, the active principle dabigatran and argatroban [158,159].

The increasing awareness of thrombin’s involvement in various pathophysiological CNS conditions suggests a potential therapeutic implication. Different pharmacological substances that interfere with thrombin activity and production are available. Vitamin-K antagonists (e.g., warfarin) slow the activation of the coagulation cascade, while heparin acts as a molecular bridge between AT III and thrombin and thus increases the inhibitory potential of AT III. Hirudin acts as a direct thrombin inhibitor that irreversibly binds to the active site of thrombin and blocks its clotting activity [149]. More recently, new direct acting thrombin inhibitors (DTI) with a lower molecular weight, such as argatroban and dabigatran, have been introduced as alternatives to the classic anticoagulants (Figure 5) [150]. The majority of studies are focused on the validation of DTIs in the therapy and prevention of ischemia. A pilot trial, designed using argatroban for its anti-thrombotic property in secondary stroke prevention, has been completed [151,152]. So far, there has been no larger study investigating DTIs for their potential role in alleviating ischemia or haemorrhage induced neuronal loss and secondary clinical deficits.

Still, argatroban has been shown to reduce the latency time, increase the learning curve and reduce primary errors during standardized neurobehavioral testing in rats after unilateral ischemic stroke [153]. A simultaneous blockade of the thrombin and C5a-receptors had a synergistic neuroprotective effect after ICH in mice. The administration of both substances after ICH reduced the occurrence of inflammatory factors and brain oedema, thus leading to improvements in neurofunctional outcome. A small preliminary study reported a reduction of oedema and improved neurological outcome in 4 patients presenting with spontaneous ICH due to hypertension when treated with intravenous argatroban [154]. Furthermore, the use of argatroban after subarachnoid hemorrhage (SAH) in a rabbit model prevents an enhancement and prolongation of the contractile response leading to cerebral vasospasm [155]. Beside that, thrombin inhibition improved the neurological outcome after experimental SAH in rats [156].

As thrombin has been shown to worsen cognitive deficits in AD, the administration of donepezil, a reversible acetylcholinesterase inhibitor used to improve cognition and behaviour, was compared to a simultaneous treatment with donepezil and hirudin. Patients receiving the dual regiment demonstrate improvements in activities of daily living, behaviour, psychological symptoms of dementia as well as cognition compared to those treated with donepezil alone [157]. These findings might be transferrable to patients suffering from CNS disease influenced by thrombin and warrants the necessity of further trials with thrombin inhibitors in these diseases.

## 6. Conclusions

During the last decade, a rising number of reports emerged, revealing the influence of thrombin not only for our coagulation system but also for neuronal signalling, development and plasticity. With our increasing knowledge about the importance of thrombin for the maintenance of functionality of the CNS, we simultaneously discover its importance for various neuronal diseases. High concentrations of thrombin seem to contribute to the pathological processes during neurodegenerative diseases, including stroke, haemorrhage, multiple sclerosis, Alzheimer’s- and Parkinson’s disease. Even rare conditions such as HIV-related dementia seem to develop under conditions influenced by thrombin signalling. In contrast to these detrimental effects, low concentrations of thrombin and thrombin preconditioning yield the potential of rescuing cells and to induce survival of neurons and astrocytes exposed to various ischemic insults. The effects of thrombin are largely mediated via PAR-1 and, to a lesser extent, via PAR-3 and PAR-4. Convincing evidence on the distinguished role of each receptor remains scarce. While PAR-1 seems to influence infarct volume, BBB integrity, neuronal damage and motor function *in vivo*, PAR-3 and PAR-4 are increasingly linked to neurodegenerative and neuroinflammatory diseases. However, conflicting data on the extent and importance of these receptors for neuropathophysiology exists. So far, only little is known about the importance of the cerebral capability to generate thrombin from its precursor prothrombin. Under most circumstances, the origin of the observed thrombin remains elusive. This underlines the dire need of further research to determine the origin of thrombin in the CNS, in order to elucidate its signalling pathways and thus helping to identify future therapeutic targets.

## Abbreviations

β-APP: amyloid-β precursor protein
Aβ: amyloid beta
AD: Alzheimer’s disease
AT: antithrombin
BBB: blood-brain-barrier
CDK 4: Cyclin-dependent kinase 4
CINC-1: cytokine-induced neutrophil chemoattractant-1
CNS: central nervous system
DTI: direct acting thrombin inhibitors
EAE: experimental autoimmune encephalitis
ERK1: extracellular signal-regulated protein kinase 1
F: factor
GFAP: glial fibrillary acidic protein
GP: glycoprotein
GRO: growth-regulated oncogene
HIF-1α: hypoxia inducing factor 1α
HIV: human immunodeficiency virus
HIVD: HIV dementia
ICH: intracerebral haemorrhage
IL: interleukin
iNOS: inducible nitric oxide synthase
JNK: N-terminal kinase
MAPK: mitogen- activated protein kinase
MMP-9: matrix metalloproteinase-9
MS: multiple sclerosis
NFTs: neurofibrillary tangles
NMDA: *N*-methyl-d-aspartate
NO: nitric oxid
OGD: glucose deprivation
6-OHDA: 6-hydroxydopamine
PARs: protease-activated receptors
PD: Parkinson’s disease
PI 3-kinase: phosphatidylinositol 3-kinase
PKC: protein kinase C
PN-1: protease nexin-1
PTX: pertussis toxin
RAS: rat sarcoma protein
ROS: reactive oxygen species
SNpc: substantia nigra pars compacta
TNF-α: tumor necrosis factor-α
TPC: thrombin preconditioning
ZO: zonula occludens
